# Prognostic Insights into Orbital Metastases: A Comprehensive Analysis of Clinical Features and Survival Outcomes

**DOI:** 10.3390/diagnostics15192542

**Published:** 2025-10-09

**Authors:** Burak Ulas, Altan Atakan Ozcan, Feyza Alara Celikten, Omer Kaya, Ertugrul Bayram

**Affiliations:** 1 Department of Ophthalmology, Faculty of Medicine, Çukurova University, Adana 01330, Türkiye; altanoz@cu.edu.tr (A.A.O.); facelikten@cu.edu.tr (F.A.C.); 2 Department of Radiology, Faculty of Medicine, Çukurova University, Adana 01330, Türkiye; omerkaya@cu.edu.tr; 3 Department of Medical Oncology, Faculty of Medicine, Çukurova University, Adana 01330, Türkiye; ertugrulbayram@cu.edu.tr

**Keywords:** orbital metastases, primary tumor, prognosis, survival analysis, breast cancer

## Abstract

**Background/Objectives:** We aimed to evaluate the demographic characteristics, clinical findings, and survival outcomes of patients diagnosed with orbital metastasis, considering primary tumor type, age, and gender variables. **Methods:** In this observational study, demographic data, tumor localization, histopathological diagnoses, and survival times of 83 patients followed for secondary orbital metastasis at Çukurova University Ophthalmology Department between 2003 and 2023 were retrospectively reviewed. Subgroup analyses were performed according to age (<18 and ≥19), gender, and primary tumor groups. **Results:** The study included 83 patients (51 (61.4%) females and 32 (38.6%) males). The mean age at diagnosis was found to be 40.8 ± 24.6 years. A total of 24.1% of the cases were in the pediatric age group (mean age 5.9 years), and the most common tumor metastasizing to the orbit in this group was neuroblastoma (80%). In adult patients, the two most frequent tumors metastasizing to the orbit were breast cancer (33.3%) and lung cancer (14.3%). The most common clinical findings were proptosis (32.5%) and blurred vision (26.2%). Orbital metastases were observed more frequently in females than in males (61.4% vs. 38.6%). This ratio was similar in the pediatric age group (65.0% vs. 35.0%). The mean survival time after metastasis was calculated as 316.7 ± 68.6 days. Female patients had a significantly longer survival time after metastasis compared to males (mean 400.4 vs. 165.4 days; *p* = 0.037). The median survival after metastasis was 86 days for patients with breast cancer and 204 days for patients with neuroblastoma. **Conclusions:** The most common source of orbital metastases in females is breast cancer, while neuroblastoma is prominent in pediatric patients. Despite all available treatment options, the prognosis after orbital metastasis is poor; this highlights the importance of early diagnosis and a multidisciplinary approach.

## 1. Introduction

The first case of orbital metastasis was described by Horner in 1864, when he reported a case of lung cancer metastasis to the orbit [1,2]. Since then, multiple cases of orbital metastasis have been described in the literature. Orbital metastases, representing the spread of primary malignancies to the orbit, are a relatively uncommon yet significant manifestation of advanced cancer, often leading to severe ocular morbidity and impacting patient quality of life. While primary orbital tumors are diverse, metastatic lesions to the orbit typically indicate systemic disease progression and are associated with a generally poor prognosis [1,2]. Understanding the clinical characteristics, primary tumor origins, and survival patterns in patients with orbital metastases is crucial for timely diagnosis, appropriate management, and improved patient outcomes.

Previous studies have shed light on various aspects of orbital metastases, with differing prevalence rates and primary tumor distributions reported across various geographical regions. For instance, a retrospective analysis from Italy highlighted breast cancer as the most frequent primary tumor in adults, consistent with findings from other Western populations, while neuroblastoma predominates in pediatric cases [2]. Similarly, studies from China and Japan have provided valuable insights into the epidemiological and clinical profiles of orbital metastases in their respective populations, often emphasizing the role of lung cancer and other systemic malignancies [3,4]. An Egyptian study also contributed to the understanding of the spectrum of orbital metastases, further illustrating the global diversity in primary tumor types and presenting symptoms [5]. More recently, Australian data have further reinforced the complexity and varied presentation of these lesions, underscoring the need for localized as well as broader epidemiological data [6].

Despite the accumulating body of literature, comprehensive analyses that simultaneously evaluate demographic variables, diverse clinical presentations, and long-term survival outcomes across different age groups and primary tumor types within a specific regional cohort remain valuable [2]. Such detailed investigations can refine our understanding of disease progression and guide more tailored therapeutic strategies. This study aims to contribute to this knowledge by retrospectively evaluating the demographic features, clinical manifestations, and survival outcomes of cancer patients diagnosed with orbital metastasis in our tertiary referral center, with a particular focus on the influence of primary tumor type, age, and gender. In doing so, we seek to provide a more detailed understanding of the natural history and prognostic factors associated with orbital metastases, which can ultimately aid in earlier diagnosis and more effective management strategies.

## 2. Materials and Methods

This study was designed as a single-center, retrospective, observational study conducted at the Orbita-Oculoplasty Unit of Cukurova University, Department of Ophthalmology. The study period encompassed patients diagnosed with secondary orbital metastasis who were followed between January 2003 and December 2023. Written informed consent for publication of clinical images was obtained from all patients or their legal guardians/first-degree relatives.

1. Patient Cohort

A total of 83 patients diagnosed with secondary orbital metastasis during the specified period were included in the study. All patients had a histopathologically confirmed diagnosis of orbital metastasis. Exclusion criteria included patients with primary orbital tumors or those with incomplete medical records precluding comprehensive data analysis.

2. Data Collection

Retrospectively, comprehensive data were collected from the electronic medical records and physical charts of the included patients. The collected variables included:

- **Demographic data:** Age at diagnosis and gender.
- **Clinical findings:** Presenting symptoms, laterality of orbital involvement, and specific signs such as proptosis, pain, vision changes, and diplopia.
- **Tumor characteristics:** Primary tumor type, location of the orbital metastasis, and histopathological diagnosis.
- **Treatment modalities:** Information regarding systemic chemotherapy, radiotherapy, surgical intervention, or other treatments received for orbital metastasis.
- **Survival outcomes:** Overall survival time from the diagnosis of orbital metastasis until the last follow-up or death.

3. Subgroup Analyses

To facilitate detailed analysis, patients were categorized into subgroups based on key variables:

- **Age groups:** Pediatric (<18 years) and adult (≥19 years). Subgroups (0–19 years, 20–39 years, 40–59 years, 60–79 years, ≥80 years)
- **Gender:** Male and female.
- **Primary tumor type:** Major primary tumor categories, including breast cancer, lung cancer, neuroblastoma, and others, were analyzed to assess their impact on clinical presentation and survival.

4. Statistical Analysis

All collected data were entered into a dedicated database and analyzed using appropriate statistical software (e.g., SPSS version 25.0). Descriptive statistics were used to summarize demographic and clinical characteristics, presented as means ± standard deviations for continuous variables and frequencies (percentages) for categorical variables.

Comparisons between groups (e.g., gender, age groups, primary tumor types) for various clinical features were performed using the Chi-square test for categorical variables and independent samples *t*-test or Mann–Whitney U test for continuous variables, as appropriate.

Survival analysis was conducted using the Kaplan–Meier method to estimate median and mean survival times from the diagnosis of orbital metastasis. Log-rank test was employed to compare survival curves between different subgroups (gender, age groups, and primary tumor types). A *p*-value of <0.05 was considered statistically significant.

5. Ethical Considerations

The study protocol was approved by the local Institutional Review Board/Ethics Committee of Cukurova University (Approval number: 3 January 2025/151-31). Due to the retrospective nature of the study, informed consent was waived. All patient data were anonymized to ensure confidentiality and privacy throughout the study.

## 3. Results

### 3.1. Patient Demographics and Tumor Characteristics

A total of 83 patients (52 females [62.7%], 31 males [37.3%]) diagnosed with orbital metastases were included in the study. The median age at diagnosis was 41 years (range: 1–82 years). Adult patients (≥19 years) had a mean age of 40.8 ± 24.6 years, while pediatric patients (<18 years) had a mean age of 5.9 years. The mean follow-up time was 11.2 months (range 1–48 months).

**Figure 1:**
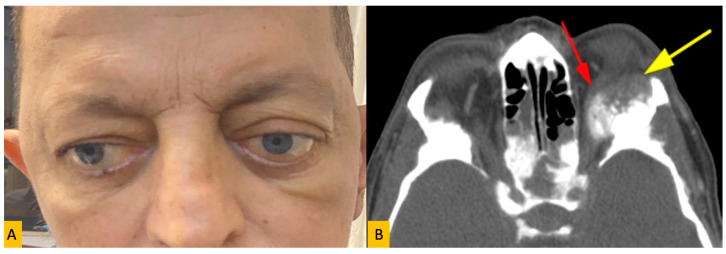
(**A**) Clinical photograph of a 49-year-old man with orbital metastasis of prostate cancer showing proptosis in the form of ptosis of the left eye. (**B**) The orbital computed tomography axial section shows a metastatic soft tissue lesion in the left orbital roof extending into the orbita and placing pressure on the superior rectus muscle (red and yellow arrows).

Of all cases, 32 patients (38.6%) had right orbital involvement, 31 (37.3%) had left orbital involvement, and 20 (24.1%) presented with bilateral disease. The most frequent clinical symptoms at presentation were proptosis (32.5%) and blurred vision (26.2%) (Figure 1).

### 3.2. Primary Tumor Distribution

**Figure 2:**
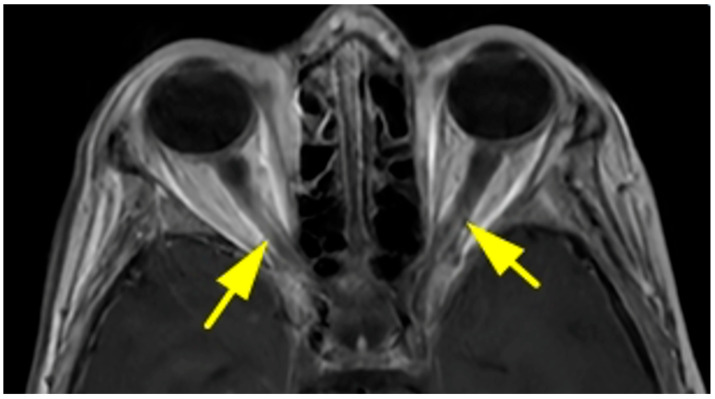
Axial T1-weighted magnetic resonance section showing bilateral optic nerve involvement in the case of a 48-year-old woman with bilateral orbital metastasis of breast cancer (yellow arrows).

**Figure 3:**
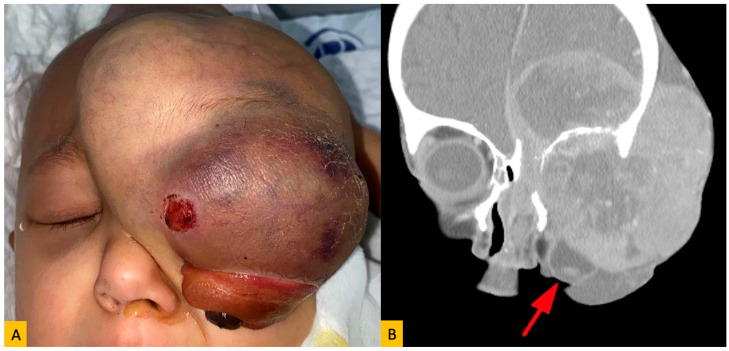
(**A**) Clinical photograph of a 4-year-old child with orbital metastasis of neuroblastoma showed proptosis, eyelid swelling and chemosis of the left eye. (**B**) Coronal computed tomography showing a massive metastatic lesion with intracranial, extracranial and intraorbital extensions in the left orbital roof, causing a protruded appearance in the left globe (red arrow) and displacing inferiorly.

The most common primary tumor was breast carcinoma (*n* = 21; 25.3%), all of which occurred in female patients (Figure 2). Lung carcinoma accounted for 10.8% (*n* = 9) of cases and was seen in both genders. Among pediatric patients, neuroblastoma was the predominant tumor type (Figure 3), with 16 cases comprising 80% of pediatric orbital metastases and 19.2% of the total cohort. Other observed primary malignancies included skin cancers (melanoma, squamous cell carcinoma, basal cell carcinoma), prostate carcinoma, bone sarcoma/myeloma, and tumors of the gastrointestinal, adrenal, and urinary tracts (Table 1). In all patients, the primary tumor diagnosis had been established prior to the detection of orbital metastasis, and there were no cases with unknown primary lesions.

### 3.3. Age Distribution and Primary Tumor Correlation

Patients were stratified into five age groups: 0–19, 20–39, 40–59, 60–79, and ≥80 years. Neuroblastoma was exclusively seen in the 0–19 age group. Breast cancer was most common in adults aged 40–59, whereas lung cancer showed a broader distribution, mainly in patients older than 40 (Table 2).

### 3.4. Survival Outcomes

**Figure 4:**
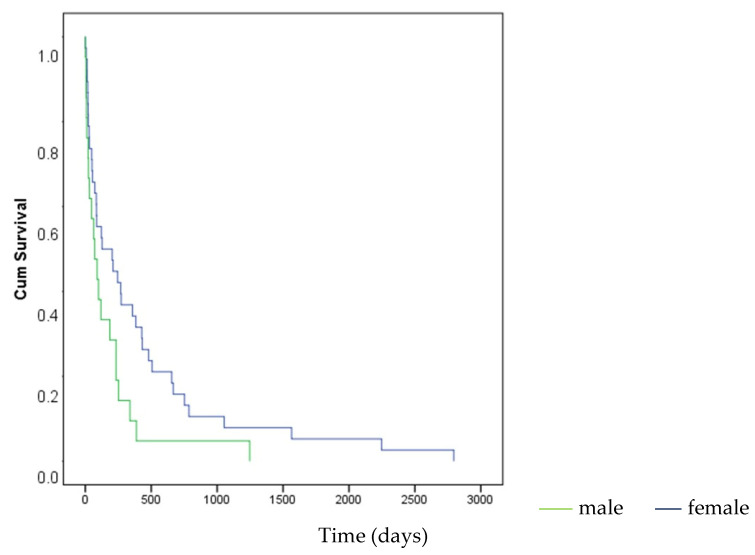
Survival time according to gender. Kaplan–Meier curve showing the female gender has a longer survival time after metastasis (in days).

The overall mean survival after diagnosis of orbital metastasis was 316.7 ± 68.6 days. Female patients exhibited significantly longer survival compared to males (mean: 400.4 vs. 165.4 days; *p* = 0.037) (Figure 4). There was no statistically significant difference in survival times between pediatric and adult patients (332.0 vs. 311.1 days; *p* = 0.473) or between different primary tumor types (*p* = 0.721). Notably, the median survival was 204 days for patients with neuroblastoma and 86 days for those with breast cancer (Table 3).

### 3.5. Time to Metastasis

The mean interval between the diagnosis of primary cancer and the development of orbital metastasis was analyzed across age groups. While the 40–59 age group had the longest average duration (6.87 years), this difference was not statistically significant (*p* = 0.664) (Table 4).

## 4. Discussion

This study provides a comprehensive analysis of orbital metastases over a 20-year period, revealing key demographic patterns, clinical features, and survival outcomes within a diverse patient population. This retrospective cohort represents one of the largest single-center series of orbital metastases in an East Mediterranean population, with an inclusion of both pediatric and adult cases over a 20-year span. Consistent with previous literature, our findings confirm that breast cancer is the most frequent primary tumor metastasizing to the orbit in adults, whereas neuroblastoma is predominant among pediatric cases [1,7]. This aligns with global trends and highlights the importance of age-specific diagnostic considerations in orbital metastatic disease [1]. Orbital metastases represent a significant clinical challenge due to their variable presentation and often poor systemic prognosis [8]. In our study of 83 patients, breast carcinoma was identified as the most common primary tumor site, followed by neuroblastoma, lung carcinoma, and bone sarcomas/myelomas. This distribution is consistent with previous reports from large-scale series, including the landmark work by Shields et al., who reported breast cancer in 53% of orbital metastasis cases, and Valenzuela et al., who noted breast and melanoma as the leading origins [1,2]. The predisposition of breast cancer may be related to estrogen produced by periorbital fat tissue [9]. As lymphatic drainage of the orbit is limited, the presence of orbital metastasis of breast cancer points out hematological spread [10].

As consistently reported in Western and Asian cohorts, breast carcinoma remains the leading cause of orbital metastases in adult females [1,4,6,7,8,9,10]. Our finding of breast cancer as the primary tumor in 33.3% of adult patients closely parallels the Italian (39%) and Australian (29%) data, while aligning with the North Chinese (25%) and Egyptian (21.6%) experience [2,3,4,5,6]. In pediatric patients, neuroblastoma emerged as the most frequent source (80%), which mirrors global reports, particularly from Egypt (42.9%) and Japan [4,5]. The age and gender distribution of orbital metastases in our study are noteworthy. The mean age at diagnosis was 40.8 ± 24.6 years. The proportion of pediatric cases (24.1%) and the predominance of neuroblastoma (80%) as the most common primary tumor metastasizing to the orbit in this group indicate a distinct etiology of orbital metastases in the pediatric population. This is consistent with data from previous international studies, such as a retrospective analysis from Italy, which highlighted the dominance of neuroblastoma in pediatric cases [2]. In adult patients, breast cancer (33.3%) and lung cancer (14.3%) were identified as the two most frequent sources of orbital metastasis. This finding is expected given the high prevalence of breast cancer in women in Western populations, including Turkiye [1,9,10,11,12,13,14,15,16,17]. Other less common primary cancers that are not specifically listed include carcinoma of the thyroid, liver, pancreas and salivary gland, choroidal melanoma, and several others. There was no thyroid cancer metastasis in our study, as in some other large series. For the most part, tumors that metastasize to the orbit are the same as those that metastasize to the uveal tract [18]. The only exception is metastasis from prostate cancer, which accounts for approximately 12% of orbital metastasis and only 2% of metastasis to the uveal tract [19]. Orbital metastases were observed more frequently in females than in males (61.4% vs. 38.6%). This ratio was similar in the pediatric age group (65.0% vs. 35.0%). This gender disparity can primarily be attributed to the higher incidence of breast cancer in women. Consistent with the Shields series, we found a female predominance, largely attributable to the high rate of breast carcinoma [1]. In our study, the primary tumor subtype of all patients diagnosed with breast cancer was carcinoma. There are limited data in the literature on histological types of breast cancer that metastasize to orbita. In a retrospective analysis of 28 cases, 14 patients had ductal, 13 patients had lobular, and 1 patient had micropapillary histology [8]. Metaplastic breast cancer is a rare malignancy that accounts for less than 1% of all types of breast carcinoma. It is known to be associated with poor prognosis and most commonly causes lung and bone metastasis [20]. Interestingly, neuroblastoma accounted for 19% of our cases and was the dominant tumor type in the pediatric population (0–19 years), aligning with findings from the Japanese and Chinese cohorts, where neuroblastoma represented a substantial fraction of childhood orbital metastases [3,4]. This underscores the importance of considering orbital metastasis in children presenting with proptosis or periorbital mass. These variations may be related to the different geographic areas of these different studies [4,5,6,7,8,9,10,11]. The actual incidence of primary tumors metastasizing to the orbit is difficult to ascertain in a clinical series compared to an autopsy series. For example, patients with bronchogenic carcinoma are usually markedly affected by their disease by the time an orbital metastasis has occurred [5].

Clinically, proptosis and blurred vision were the most common presenting signs in our cohort, as also highlighted in studies from China (91.7% proptosis), Egypt (78.4%), and Italy (73%) [2,3,4,5]. These nonspecific symptoms often mimic other orbital disorders such as thyroid orbitopathy, delaying diagnosis—a challenge echoed across all referenced studies. Extraocular muscle disturbance that occurs in our series in 23,8% can be due to direct tumor infiltration of the muscle or to mass effect or to nerve palsies; rarely it develops as part of a paraneoplastic phenomenon [17]. Notably, diplopia, ptosis, and pain were more prevalent in Asian cohorts, potentially reflecting differences in tumor location or patient delay in seeking care [3,4]. The consistency in clinical presentation across different studies emphasizes the importance of these signs for early detection [12,15].

The site and extent of orbital involvement also varied by population. While our study did not analyze quadrant localization in detail, the Chinese cohort reported a predominance in the superior orbit, while the Australian group noted fat and muscle infiltration as common imaging findings [3,6]. Bone invasion was strongly associated with prostate and HCC metastases in the Egyptian and Italian reports [2,5]. The diagnostic and therapeutic approaches across studies remain similar. All series, including ours, employed a combination of clinical evaluation, orbital imaging, and histopathological confirmation [3,9,13,14,15]. Treatment was largely palliative and multimodal—radiotherapy, chemotherapy, and occasionally surgery—aimed at symptom control rather than curative intent [9]. Despite such efforts, prognosis remains poor [2,3,4,5,6,7,8,9,10]. The overall survival across studies seldom exceeded 1–1.5 years, emphasizing the need for early recognition and coordinated oncologic care.

The age distribution in our cohort revealed that most metastases occurred in middle-aged and elderly individuals, similar to other international series [4,6,7,8,9]. However, the presence of patients younger than 20 years, particularly with adrenal-origin tumors, highlights the need for age-specific diagnostic considerations. The median time from diagnosis of the primary tumor to orbital metastasis varied significantly across age groups. In adults, particularly those aged 60–79, the median interval was 7 years, which is slightly longer than the median interval reported by Shields et al. (55 months) [1]. Pediatric patients had a much shorter interval, suggesting more aggressive disease behavior or earlier detection due to systemic evaluation prompted by other symptoms.

In the present study, survival analysis revealed a mean survival time of 316.7 ± 68.6 days after metastasis, underscoring the generally poor prognosis associated with orbital metastases. A significant finding was the longer survival time in female patients compared to males (mean 400.4 vs. 165.4 days; *p* = 0.037). This gender-related survival advantage could be linked to the prevalence of specific primary tumor types in females, particularly breast cancer, which may have a slower progression or be more amenable to certain systemic therapies compared to, for example, lung cancer in males [9]. The Italian study reported a median overall survival of 10 months [9]. The Chinese study reported a median survival time of 8 months, while the Japanese study indicated a median survival of 7.9 months [3,4]. The Egyptian study found a median survival of 6 months [5]. The Australian study, with a median survival of 7 months, also highlighted the grim prognosis [6]. A recent retrospective cohort from Thailand further broadened the perspective by evaluating intraocular and adnexal metastases, reporting overall survival rates comparable to orbital cases. The authors emphasized that ocular metastatic involvement, regardless of location, consistently indicates advanced systemic disease and poor prognosis [21]. A recent bi-institutional study from China demonstrated that metastatic ocular and orbital melanoma carry a poor prognosis, with a median overall survival of only 11.9 months despite multimodal therapies. The authors also highlighted that liver metastases were particularly common in uveal melanoma patients, underscoring the aggressive nature of the disease [22]. Our mean survival of approximately 10.5 months (316.7 days) falls within the range reported by these international studies, confirming the aggressive nature of this disease manifestation [3,4,5,6,7,8,9]. While our study found no significant difference in survival between age groups (*p* = 0.473) and primary tumor types (*p* = 0.721), it is important to note that the median survival after metastasis was 86 days for breast cancer and 204 days for neuroblastoma in our cohort. Some international studies have indicated that certain primary tumor types, such as neuroblastoma or prostate cancer, might be associated with slightly better prognoses compared to lung cancer or melanoma [7,8]. However, the overall consensus remains that orbital metastasis signifies advanced systemic disease with a limited life expectancy [1]. Variability in survival data across studies likely stems from differences in systemic disease burden, cancer subtypes, and treatment availability. Survival outcomes in our study were influenced by gender, with female patients demonstrating significantly longer post-metastasis survival than males (median 127 vs. 70 days, *p* = 0.037). Although this gender-related survival difference has not been consistently emphasized in previous studies, it may reflect tumor biology or treatment disparities. Breast cancer patients, in particular, tended to have longer survival times, a finding also noted by Shields et al. and others [1].

Interestingly, neuroblastoma patients exhibited relatively prolonged survival (median 204 days) compared to other tumor types, potentially attributable to advances in pediatric oncology and aggressive multimodal therapy. In contrast to our study, the survival time after metastasis in neuroblastoma was found to be aggressively short in the Chinese study [3]. However, as also pointed out in the Egyptian series, survival times remained limited in most metastatic cases, reinforcing the overall poor prognosis associated with orbital involvement [5].

Most of the orbital metastases presented in patients with known primary tumors, probably because of an increasing awareness and advances in medicine for early cancer detection [1,10]. To give an example, Vlachostergios et al. reported a lag of 4.5–6.5 years between initial diagnosis and the onset of symptoms and found that up to 25% of patients with metastatic disease do not carry a known primary at the time of diagnosis [23]. Alternatively, a review by Garrity et al. reported that 76% of orbital metastases occurred in patients over the age of 75 and that 19–25% of these patients did not have a known history of malignancy [19]. In contrast, there were no patients in our study without a primary cancer diagnosis. In our cohort, although this proportion was lower than the 19% reported by Shields et al., it underscores the ophthalmologist’s critical role in the initial diagnosis and systemic referral [1]. Genetic predisposition analysis could not be performed due to the retrospective design of the study and the fact that the majority of patients included in the study were not alive.

The strengths of our study include its comprehensive data collection spanning two decades, providing valuable insights into long-term outcomes within a specific regional cohort. However, limitations, such as a single-center, retrospective design and relatively moderate sample size may limit generalizability. The other limitations of our study include its retrospective design, lack of detailed systemic disease staging, and limited data on treatment regimens and molecular subtypes. Another limitation of our study is the lack of a detailed multivariate survival analysis, since systemic treatments and pathological subtypes were heterogeneous and not uniformly documented across the cohort. Due to the retrospective design and incomplete uniform documentation across the 20-year period, individual patient-level data could not be presented as a supplementary table. Nevertheless, our analysis adds to the global literature by offering comparative insights from a tertiary center in Turkey, encompassing both pediatric and adult cases with confirmed histopathology. Despite these limitations, our findings contribute important regional epidemiological data to the growing global understanding of orbital metastases.

## 5. Conclusions

In conclusion, this study provides valuable information on the clinical spectrum, primary tumor sources, and survival prognosis of orbital metastases within a Turkish population. It reinforces that breast cancer is the most common primary tumor in adult females, while neuroblastoma predominates in pediatric patients, aligning with global trends. The finding of gender being an important factor affecting survival time, with females demonstrating longer survival, warrants further investigation into underlying biological and treatment-related factors. Given the consistently poor prognosis reported across our study and the international literature, the paramount importance of early diagnosis and a multidisciplinary approach for optimizing patient management is underscored. Regional studies like ours remain crucial to contextualizing global patterns and optimizing multidisciplinary care strategies for orbital metastasis. Future research focusing on molecular profiling, targeted therapies, and larger multi-center studies will be crucial to further refine our understanding and improve outcomes for patients with orbital metastases.

## Figures and Tables

**Table 1:** Distribution of primary tumor sites causing orbital metastases by histological type and gender.

Primary Site	Tumor Type	Number of Patients	% of Total	Male (*n*)	Female (*n*)
Breast	Carcinoma	21	25.3%	0	21
Prostate Gland	Carcinoma	3	3.6%	3	0
Lung	Carcinoma	9	10.8%	5	4
Skin	Melanoma (*n* = 4), SCC (*n* = 2), BCC (*n* = 1)	7	8.4%	3	4
Adrenal Gland	Neuroblastoma	16	19.2%	5	11
Gastrointestinal Tract	Carcinoma	3	3.6%	2	1
Urinary Tract	Carcinoma	2	2.4%	2	0
Bone	Sarcoma (*n* = 5), Myeloma (*n* = 5)	10	12.0%	6	4

Abbreviations: SCC = Squamous Cell Carcinoma; BCC = Basal Cell Carcinoma.

**Table 2:** Age distribution of patients according to primary tumor site.

Primary Site	0–19 Years	20–39 Years	40–59 Years	60–79 Years	≥80 Years
Breast	0	5	9	6	1
Prostate Gland	0	0	1	2	0
Lung	0	1	2	6	0
Skin	0	0	4	1	2
Adrenal Gland	16	0	0	0	0
Gastrointestinal Tract	0	0	1	2	0
Urinary Tract	0	0	1	1	0
Bone	2	2	3	3	0

**Table 3:** Survival times after diagnosis of orbital metastasis according to gender, age group, and primary tumor type (in days).

Category	Subgroup	Mean Survival (Day)	Median Survival (Day)	*p*-Value
**Gender**	Female	400.4	127.0	0.037
	Male	165.4	70.0	
**Age Group**	<18 years	332.0	245.0	0.473
	≥19 years	311.1	80.0	
**Primary Tumor**	Breast	383.0	86.0	0.721
	Neuroblastoma	338.0	204.0	
	Others	358.0	70.0	

Note: *p*-values were calculated using the log-rank test.

**Table 4:** Time interval from primary tumor diagnosis to orbital metastasis by age group (in years).

Age Group	Mean Time to Metastasis (Year)	Median Time to Metastasis (Year)	*p*-Value
0–19 years	1.03	2.0	0.664
20–39 years	3.40	4.0	
40–59 years	6.87	3.0	
60–79 years	5.09	7.0	
≥80 years	1.66	2.0	

Note: ‘Time to metastasis’ refers to the interval between primary tumor diagnosis and the development of orbital metastasis.

## Data Availability

The original contributions presented in this study are included in the article. Further inquiries can be directed to the corresponding author.
